# Variable screening and model construction for prognosis of elderly patients with lower-grade gliomas based on LASSO-Cox regression: a population-based cohort study

**DOI:** 10.3389/fimmu.2024.1447879

**Published:** 2024-09-11

**Authors:** Xiaodong Niu, Tao Chang, Yuekang Zhang, Yanhui Liu, Yuan Yang, Qing Mao

**Affiliations:** Department of Neurosurgery, Neurosurgery Research Laboratory, and West China Glioma Center, West China Hospital, Sichuan University, Chengdu, Sichuan, China

**Keywords:** cancer-specific survival, elderly, lower-grade glioma, nomogram, overall survival, SEER

## Abstract

**Background:**

This study aimed to identify prognostic factors for survival and develop a prognostic nomogram to predict the survival probability of elderly patients with lower-grade gliomas (LGGs).

**Methods:**

Elderly patients with histologically confirmed LGG were recruited from the Surveillance, Epidemiology, and End Results (SEER) database. These individuals were randomly allocated to the training and validation cohorts at a 2:1 ratio. First, Kaplan−Meier survival analysis and subgroup analysis were performed. Second, variable screening of all 13 variables and a comparison of predictive models based on full Cox regression and LASSO-Cox regression analyses were performed, and the key variables in the optimal model were selected to construct prognostic nomograms for OS and CSS. Finally, a risk stratification system and a web-based dynamic nomogram were constructed.

**Results:**

A total of 2307 elderly patients included 1220 males and 1087 females, with a median age of 72 years and a mean age of 73.30 ± 6.22 years. Among them, 520 patients (22.5%) had Grade 2 gliomas, and 1787 (77.5%) had Grade 3 gliomas. Multivariate Cox regression analysis revealed four independent prognostic factors (age, WHO grade, surgery, and chemotherapy) that were used to construct the full Cox model. In addition, LASSO-Cox regression analysis revealed five prognostic factors (age, WHO grade, surgery, radiotherapy, and chemotherapy), and a LASSO model was constructed. A comparison of the two models revealed that the LASSO model with five variables had better predictive performance than the full Cox model with four variables. Ultimately, five key variables based on LASSO-Cox regression were utilized to develop prognostic nomograms for predicting the 1-, 2-, and 5-year OS and CSS rates. The nomograms exhibited relatively good predictive ability and clinical utility. Moreover, the risk stratification system based on the nomograms effectively divided patients into low-risk and high-risk subgroups.

**Conclusion:**

Variable screening based on LASSO-Cox regression was used to determine the optimal prediction model in this study. Prognostic nomograms could serve as practical tools for predicting survival probabilities, categorizing these patients into different mortality risk subgroups, and developing personalized decision-making strategies for elderly patients with LGGs. Moreover, the web-based dynamic nomogram could facilitate its use in the clinic.

## Introduction

Diffuse gliomas are categorized into three grades (grades 2, 3, and 4) based on histopathological and molecular characteristics according to the classification guidelines for central nervous system (CNS) tumors (1, 2). Among diffuse gliomas, glioblastoma (GBM) is the most prevalent histological type, accounting for more than half of all gliomas (3, 4). Additionally, lower-grade gliomas (LGGs) include Grade 2 and 3 (called Grade II and III previously) gliomas, which are distinguished by similar mitotic activity, mutations in isocitrate dehydrogenase (IDH), and survival outcomes characterized by specific molecular features that distinguish them from GBM (5–7). LGGs can occur in all age groups, and the peak incidence of astrocytoma occurs in the fourth decade of life, whereas oligodendrogliomas typically affect a slightly older population (8, 9). Moreover, elderly patients with LGGs typically exhibit poorer survival outcomes due to increasing medical comorbidities and diminished physiological reserves, making their clinical management challenging.

To our knowledge, several predictive models have been developed for patients with GBMs or high-grade gliomas in middle-aged and older populations (10–13). Although previous studies have identified various prognostic factors for overall survival in LGG patients (14, 15), little is known about the prognostic factors influencing survival in elderly patients with LGG, and no specific clinical prognostic model tailored to this population has been established. Given the limited inclusion of older data in prior studies, developing a prognostic model dedicated to elderly patients is imperative. In this investigation, we aimed to extract and analyze data from elderly LGG patients with large sample sizes from the Surveillance, Epidemiology, and End Results (SEER) database to determine independent prognostic factors for overall survival (OS) and cancer-specific survival (CSS) based on different regression methods. Subsequently, we constructed a novel nomogram and risk stratification system, which could help clinicians quickly develop clinical assessments and personalized decision-making strategies for elderly patients with LGGs.

## Materials and methods

### Study population and data collection

**Figure 1:**
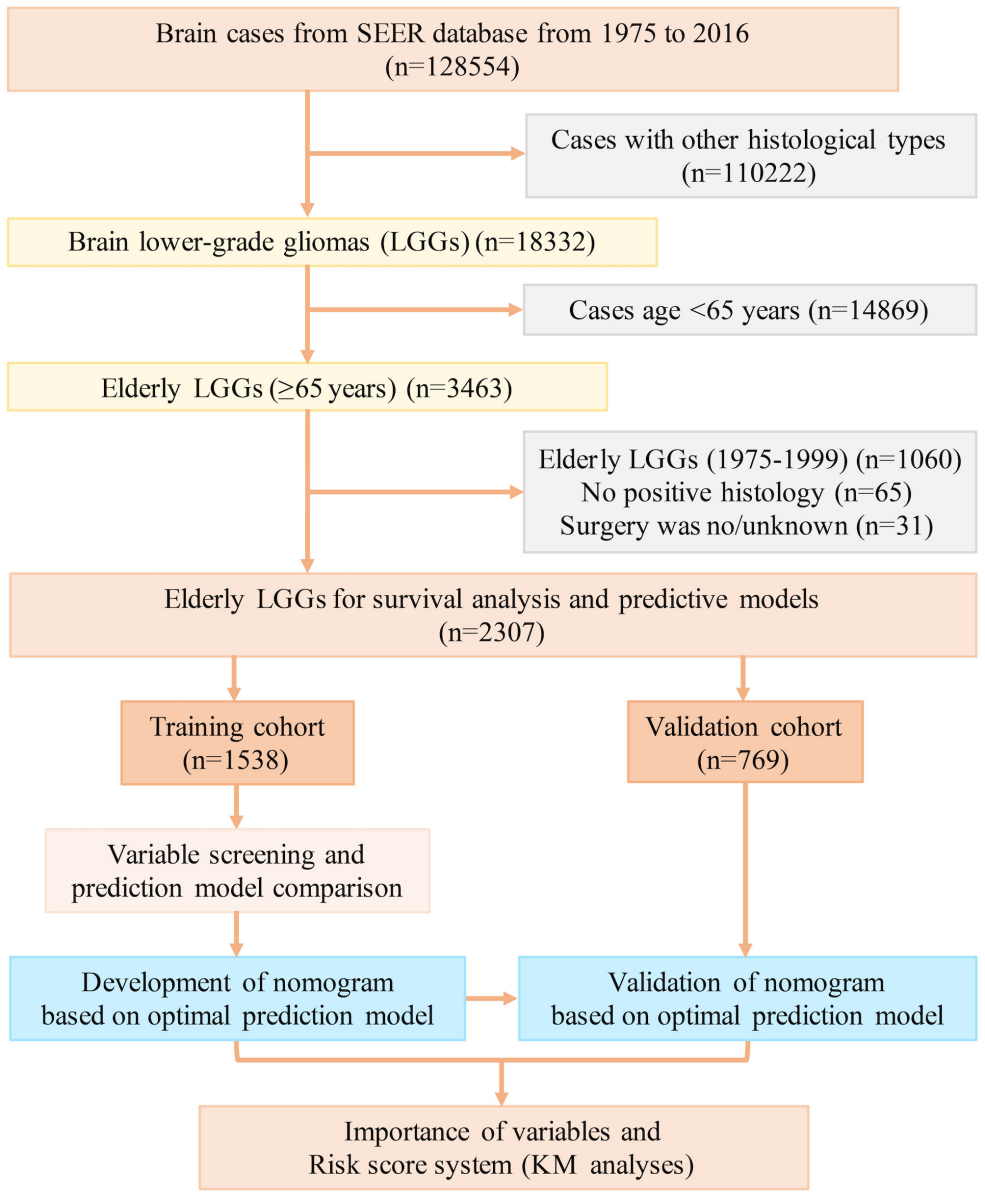
The flow diagram of case inclusion and exclusion. LGG, lower-grade glioma; SEER, Surveillance, Epidemiology, and End Results.

Elderly lower-grade glioma (LGG) patients (aged over 65 years) were identified from the Surveillance, Epidemiology, and End Results (SEER) database (17 registries) spanning from 2000 to 2016 using SEER*Stat 8.3.6 software. LGGs included oligodendroglioma, diffuse astrocytoma (DA), anaplastic oligodendroglioma (AO), and anaplastic astrocytoma (AA) on the basis of the International Classification of Diseases for Oncology, third edition (ICD-O-3). The inclusion criteria were as follows: 1) confirmed diagnosis of LGG; 2) aged older than 65 years; and 3) availability of key clinical and survival data (including age, sex, surgical interventions, survival duration, and outcomes) from the SEER database. Patients lacking the abovementioned critical clinical data were excluded. A flowchart detailing the case inclusion and exclusion criteria is illustrated in **Figure 1**. A total of 2307 elderly patients with LGGs from the SEER database were enrolled in this study. These individuals were randomly divided into training and validation cohorts at a ratio of 2:1 to establish and validate the prognostic nomograms for OS and CSS. This study was reported in line with the Strengthening the Reporting of Cohort Studies in Surgery (STROCSS) criteria (16). This study involving humans was approved by the ethics committee of the West China Hospital, Sichuan University.

### Variables of interest

The characteristics of elderly LGG patients were outlined based on key variables of interest, including patient demographics (age, sex, race, insurance coverage, and year of diagnosis), tumor attributes (location, size, stage), treatment modalities (surgery type, radiotherapy, chemotherapy), survival time (months), and vital status for an individual patient. These variables were extracted from the SEER database and further categorized according to the database codes. Race was divided into White, Black, and other groups (comprising American Indian/AK Native, and Asian/Pacific Islander). Age was stratified into three groups: 65-69, 70-79, and ≥80 years. The year of diagnosis was categorized into two intervals: 2000-2009 and 2010-2016. The tumor location was recorded as unilateral or bilateral/midline. The tumor site was specified as the frontal or nonfrontal lobes. Tumor stage was defined as local or invasive/distant involvement. The median tumor size served as the size cutoff. Tumor grade was classified into grade 2 (oligodendroglioma and DA) and grade 3 (AO and AA). The surgical type or extent of resection (EOR) was documented as gross total resection (GTR), subtotal resection (STR), partial resection (PR), or biopsy (refer to the SEER Program Coding and Staging Manual 2024, Appendix C: Surgery Codes). OS was defined as the time interval between the initial diagnosis and mortality from any cause. CSS is defined as the time interval between the initial diagnosis and mortality from cancer-related causes. OS and CSS were defined as the primary endpoints of this study.

### Kaplan−Meier survival analysis

Kaplan−Meier (K−M) survival analysis with a log-rank test was employed to evaluate the impact of variables of interest on OS. Univariate and multivariate Cox regression analyses were performed to calculate hazard ratios (HRs) with corresponding 95% confidence intervals (CIs) and to identify independent prognostic factors for OS and CSS in the training cohort. A *P* value less than 0.05 for these variables was considered to indicate statistical significance.

### Variable screening and model selection

In this study, a total of 13 variables were included. First, all 13 variables were included in the model (Model 1). Furthermore, univariate Cox regression was used to screen the variables with significant differences in survival, which were then included to determine the independent prognostic factors via multivariate Cox regression and to construct a full Cox model (Model 2). Additionally, LASSO regression is a novel method for variable screening that is based on the Cox model (LASSO-Cox regression) by applying a penalized regression on all variable coefficients (16). Lambda.1se represents the optimal lambda (λ) for screening the variables and was used to construct the LASSO model (Model 3). The above three predictive models in the training cohort were evaluated via Harrell’s concordance index (C-index) and area under the curve (AUC) values of receiver operating characteristic (ROC) curves. The optimal predictive model was further used to construct a prognostic nomogram for visualization.

### Development and validation of the prognostic nomogram

Variables included in the optimal predictive model were incorporated to construct prognostic nomograms for predicting 1-year, 2-year, and 5-year OS and CSS in elderly patients with LGGs. Next, we validated the nomogram’s predictive ability and clinical utility with internal and external validation consisting of 1000 bootstrap resamples of the models. Discrimination and reliability were assessed by computing the C-index and AUC values. Higher C-index and AUC values indicated superior discrimination ability (range: 0.5-1.0). Calibration plots were generated to verify the consistency between the predicted and observed values. Decision curve analysis (DCA) curves were generated to assess the clinical utility of the prognostic nomograms. Additionally, the importance of variables in the prognostic nomogram model was evaluated via the random forest model and machine learning. Finally, a risk stratification system based on the nomograms and a web-based dynamic nomogram was constructed.

### Statistical analysis

Continuous variables are presented as the mean ± standard deviation, and comparisons were made using t-tests and chi-squared tests for continuous and categorical variables, respectively. K−M curves with log-rank tests were utilized to assess the influence of variables on OS. Cox regression and LASSO regression analyses were conducted to determine the independent prognostic factors for OS and CSS. Lambda.1se represented the optimal lambda (λ) for screening the variables. The median total score of the nomogram was defined as the cutoff value of the risk score. The importance of variables and prognostic nomograms were performed using R software packages, including “survival”, “rms”, “regplot”, “survivalROC”, “ggDCA”, “glmnet”, “randomForest”, “xgboost”, “shapviz”, “nomogramFormula”, and “DynNom”. A two-sided *P* value <0.05 was considered to indicate statistical significance. Statistical analyses were performed using R (version 4.2.2) software.

## Results

### Patient demographics and clinical characteristics

A total of 2307 elderly patients with LGGs were enrolled in this study. Among these patients, 1220 were male (52.9%) and 1087 were female (47.1%). The median age was 72 years, with a mean age of 73.30 ± 6.22 years. The age distribution consisted of 789 (34.2%) patients aged 65-69 years, 1094 (47.4%) aged 70-79 years, and 424 (18.4%) aged ≥80 years. The majority of patients were White (2097, 90.4%), followed by Black (104, 4.7%) and other racial groups (106, 4.9%). The most prevalent tumor site was the frontal lobe (679, 29.4%). Patients were categorized based on tumor size: <38 mm (724, 31.4%) and ≥38 mm (735, 31.9%) subgroups. The majority of patients exhibited local tumor involvement (2208, 95.7%), with a small fraction showing invasive/distant tumor extension (25 patients, 1.1%). According to the World Health Organization (WHO) grade, 520 (22.5%) patients had Grade 2 gliomas, while 1787 (77.5%) had Grade 3 gliomas. The surgical resection types included GTR/STR (394, 17.1%), PR/biopsy (867, 37.5%), and no surgery (1046, 45.4%). Radiotherapy was administered to 803 (34.8%) patients, and chemotherapy was administered to 989 (42.9%) patients. Adjuvant therapy was stratified into the following subgroups: chemoradiotherapy (534, 23.2%), radiotherapy only (269, 11.6%), chemotherapy only (455, 19.7%), and no adjuvant therapy (1049, 45.5%). All enrolled patients were randomly divided into a training cohort (n=1538, 66.7%) and a validation cohort (n=769, 33.3%) for the development and validation of prognostic nomograms, with no significant intergroup differences observed (P>0.05). **Table 1** presents the population characteristics and clinical features of both cohorts.

**Table 1:** Summary of clinicopathologic features and treatments of elderly patients with LGGs.

Variables	Training cohort	Validation cohort	Overall
No.	1538	769	2307
Age at diagnosis (years)			
Mean	73.32 ± 6.12	73.24 ± 6.40	73.30 ± 6.22
Median	73	72	72
65-69	510 (33.2%)	279 (36.3%)	789 (34.2%)
70-79	748 (48.6%)	346 (45.0%)	1094 (47.4%)
≥80	280 (18.2%)	144 (18.7%)	424 (18.4%)
Sex			
Male	823 (53.5%)	397 (51.6%)	1220 (52.9%)
Female	715 (46.5%)	372 (48.4%)	1087 (47.1%)
Race			
White	1406 (91.4%)	681 (88.7%)	2087 (90.4%)
Black	63 (4.1%)	43 (5.5%)	106 (4.7%)
Other	69 (4.5%)	45 (5.8%)	114 (4.9%)
Year at diagnosis			
2000-2009	868 (56.4%)	442 (57.4%)	1310 (56.8%)
2010-2016	670 (43.6%)	327 (42.6%)	997 (43.2%)
Insurance			
Yes	824 (53.6%)	420 (54.6%)	1244 (53.9%)
No/Unknown	714 (46.4%)	349 (45.4%)	1063 (46.1%)
Tumor side			
Unilateral	221 (14.4%)	132 (17.1%)	353 (15.3%)
Bilateral/Midline	19 (1.2%)	9 (1.2%)	28 (1.2%)
Unknown	1298 (84.4%)	628 (81.7%)	1926 (83.5%)
Tumor site			
Frontal	443 (28.8%)	236 (30.7%)	679 (29.4%)
Nonfrontal	661 (43.0%)	318 (41.4%)	979 (42.5%)
Unknown	434 (28.2%)	215 (27.9%)	649 (28.1%)
Tumor size (mm)			
<38	480 (31.2%)	244 (31.7%)	724 (31.4%)
≥38	492 (32.0%)	243 (31.6%)	735 (31.9%)
Unknown	566 (36.8%)	282 (36.7%)	848 (36.7%)
Tumor stage			
Local	1470 (95.6%)	738 (95.9%)	2208 (95.7%)
Invasive/Distant	17 (1.1%)	8 (1.1%)	25 (1.1%)
Unknown	51 (3.3%)	23 (3.0%)	74 (3.2%)
WHO grade			
Grade 2	344 (22.4%)	176 (22.8%)	520 (22.5%)
Grade 3	1194 (77.6%)	593 (77.2%)	1787 (77.5%)
Surgery (EOR)			
GTR/STR	268 (17.4%)	126 (16.4%)	394 (17.1%)
PR/Biopsy	582 (37.9%)	285 (37.1%)	867 (37.5%)
No	688 (44.7%)	358 (46.5%)	1046 (45.4%)
Radiotherapy			
Yes	531 (34.5%)	272 (35.4%)	803 (34.8%)
No/unknown	1007 (65.5%)	497 (64.6%)	1504 (65.2%)
Chemotherapy			
Yes	651 (42.3%)	338 (44.0%)	989 (42.9%)
No/unknown	887 (57.7%)	431 (56.0%)	1318 (57.1%)
Adjuvant therapy			
Radio only	181 (11.7%)	88 (11.4%)	269 (11.6%)
Chemo only	301 (19.6%)	154 (20.0%)	455 (19.7%)
Radio + Chemo	350 (22.8%)	184 (23.9%)	534 (23.2%)
No	706 (45.9%)	343 (44.6%)	1049 (45.5%)
Median OS (months)			
Overall	6	6	6
Grade 2	15.0	13.0	14.5
Grade 3	5	5	5

No., number; EOR, extent of resection; GTR, gross total resection; STR, subtotal resection; PR, partial resection; Radio, radiotherapy; Chemo, chemotherapy; OS: overall survival.

### Kaplan−Meier survival analysis

**Figure 2:**
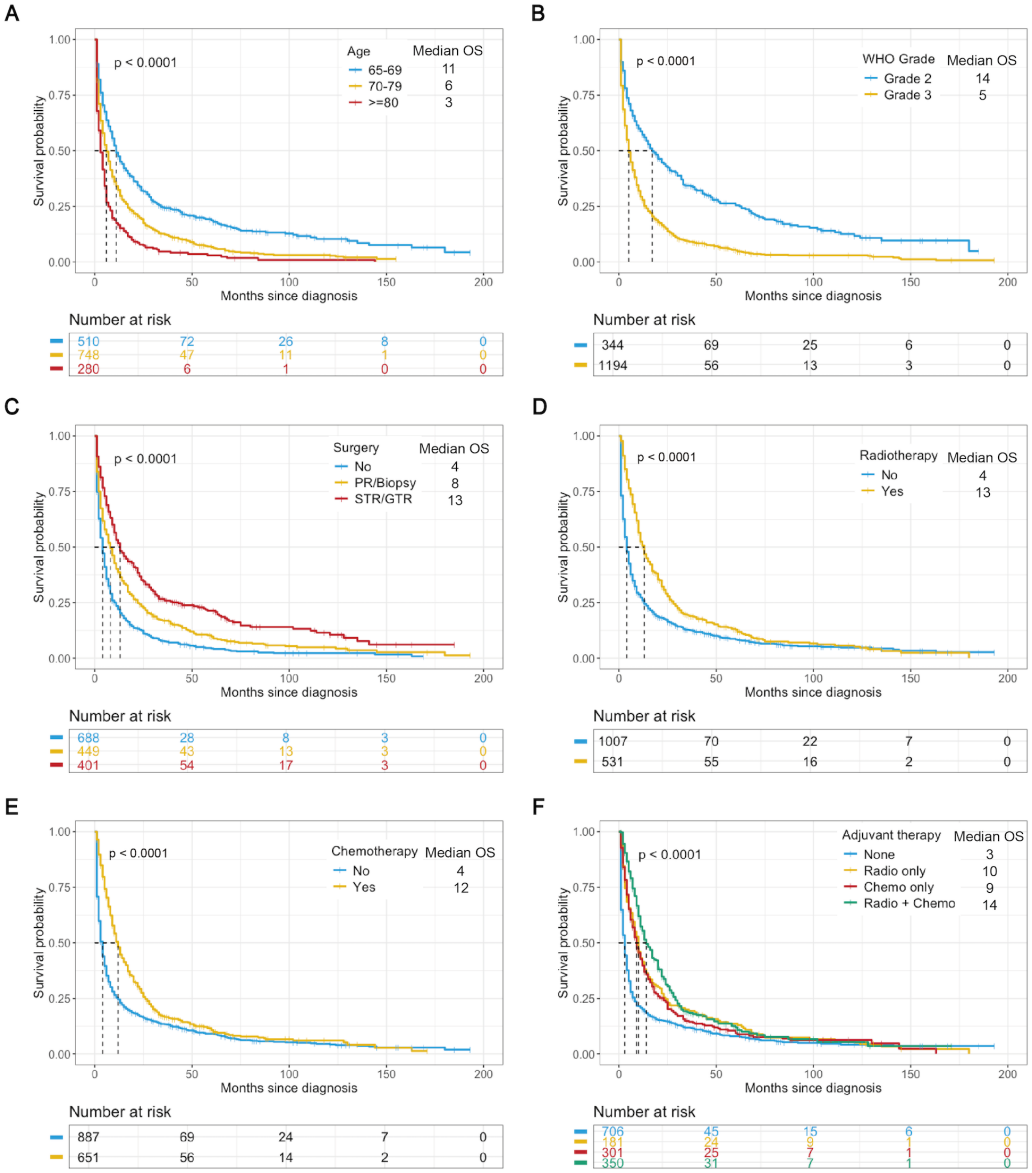
K−M analysis determining the impact of variables on OS (months) in the training cohort. Stratified by age **(A)**, WHO grade **(B)**, surgery **(C)**, radiotherapy **(D)**, chemotherapy **(E)**, and adjuvant therapy **(F)**. Chemo, chemotherapy; Radio, radiotherapy.

All elderly patients with LGGs had a median OS of 6.0 months and a mean OS of 16.2 months. When stratified by WHO grade, patients with Grade 2 gliomas (22.5%) had longer OS than did those with Grade 3 gliomas (77.5%), with median OS of 14.5 months and 5 months, respectively (**Table 1**). K−M analysis was used to determine the impact of variables on OS in the training cohort. Variables such as younger age, grade 2 tumors, maximal resection (GTR/STR), and adjuvant therapy (radiotherapy and chemotherapy) were associated with better survival outcomes (**Figure 2**). According to subgroup analyses based on WHO grade, different treatment modalities had varying impacts on OS. Notably, Grade 2 patients who underwent maximal resection without adjuvant chemoradiotherapy had the most favorable survival outcomes **(** **Supplementary Figure 1**), whereas Grade 3 patients benefitted more from surgical resection combined with adjuvant therapy (**Supplementary Figure 2**).

### Cox regression and LASSO regression analysis

**Figure 3:**
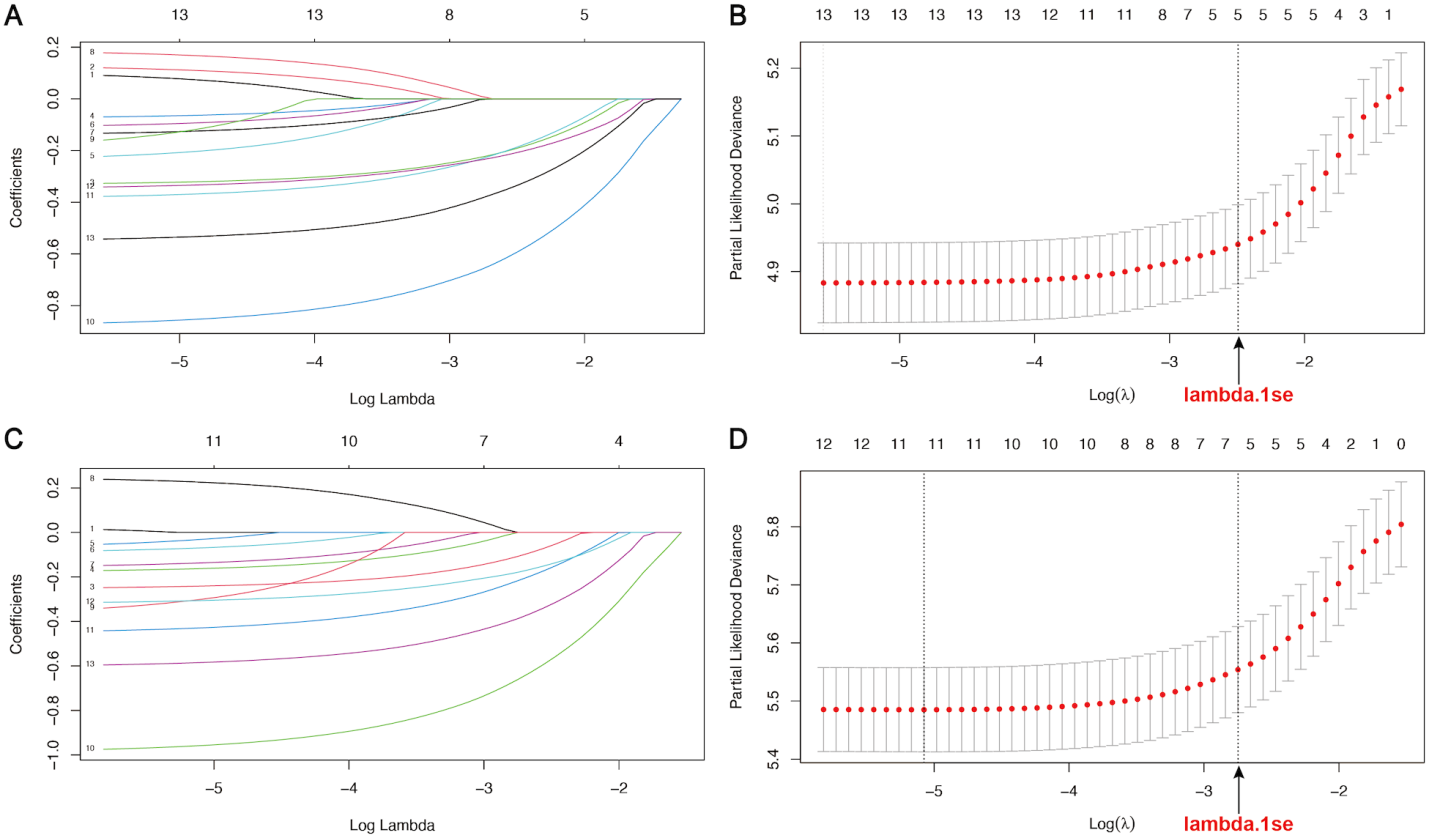
LASSO regression curves of variables in the training cohort. **(A, B)** The curve of the regression coefficient versus log (λ) and the partial likelihood deviation versus log (λ) for OS. **(C, D)** The curve of the regression coefficient versus log (λ) and the partial likelihood deviation versus log (λ) for CSS. Lambda.1se represents the optimal lambda (λ) for screening the variables.

Univariate and multivariate Cox regression analyses identified 4 variables (age, WHO grade, surgery, and chemotherapy) as independent prognostic factors for OS and CSS (**Supplementary Table 1**). In addition, we used LASSO-Cox regression to screen for variables significantly associated with OS and CSS. The curves of the regression coefficient versus log (λ) and the partial likelihood deviation versus log (λ) were used to screen 5 independent prognostic factors associated with OS and CSS (age, WHO grade, surgery, radiotherapy, and chemotherapy) when lambda.1se represented the optimal lambda (**Figure 3**). Multivariate Cox regression analyses showed that these five variables (*P*<0.05) were independent prognostic factors for OS and CSS. On the basis of the above, we constructed three predictive models (Models 1, 2, and 3) according to the number of variables based on the different screening methods. Three predictive models were compared by using the C-index (**Table 2**) and AUC values (**Table 3**). The results showed that Model 3 (LASSO model, five variables) had higher C-index and AUC values than Model 2 (four variables) and had the same predictive power as Model 1 (all variables). Thus, we decided that the five variables (age, WHO grade, surgery, radiotherapy, and chemotherapy) in Model 3 (the optimal predictive model) were further utilized to construct the prognostic nomogram.

**Table 2:** The comparison of C-index of different models in the training cohort.

Comparison of models	Model 1	Model 2	Model 3
**Methods of variable screen**	All variables	Full Cox	LASSO-Cox
Analysis for OS			
No. of variables	13	4	5
C-index (95% CI)	0.709 (0.694-0.724)	0.704 (0.689-0.718)	0.710 (0.695-0.725)
*P* Value (*vs.* Model 1) **^a^**	–	1.971e-05 ***	5.642e-05 ***
*P* Value (*vs.* Model 2) **^a^**	–	–	0.03406 *
Analysis for CSS			
No. of variables	13	4	5
C-index (95% CI)	0.707 (0.689-0.724)	0.700 (0.682-0.717)	0.705 (0.687-0.723)
*P* Value (*vs.* Model 1) **^a^**	–	0.005769 **	0.003562 **
*P* Value (*vs.* Model 2) **^a^**	–	–	0.556

**^a^** DeLong tests. Model 1, including all 13 variables; Model 2, including 4 variables screened by univariate and multivariate COX regression analysis; Model 3, including 5, screened by LASSO-Cox regression analysis. CI, confidence interval; C-index, Harrell’s concordance index; OS: overall survival; CSS: cancer-specific survival; ***, < 0.001; **, <0.01; *, <0.05.

**Table 3:** The comparison of AUC values of three models in the training cohort.

Comparison of models		Model 1	Model 2	Model 3
Analysis for OS				
No. of variables		13	4	5
AUC (1-year)		0.782	0.772	0.775
AUC (2-year)		0.785	0.771	0.772
AUC (5-year)		0.822	0.806	0.805
Analysis for CSS				
No. of variables		13	4	5
AUC (1-year)		0.775	0.762	0.768
AUC (2-year)		0.782	0.758	0.762
AUC (5-year)		0.806	0.775	0.787

Model 1, including all 13 variables; Model 2, including 4 variables screened by univariate and multivariate Cox regression analysis; Model 3, including 5 variables screened by LASSO-Cox regression analysis. AUC, Area under the ROC curve; OS: overall survival; CSS: cancer-specific survival.

### Interpreting the importance of variables in the prognostic model

**Figure 4:**
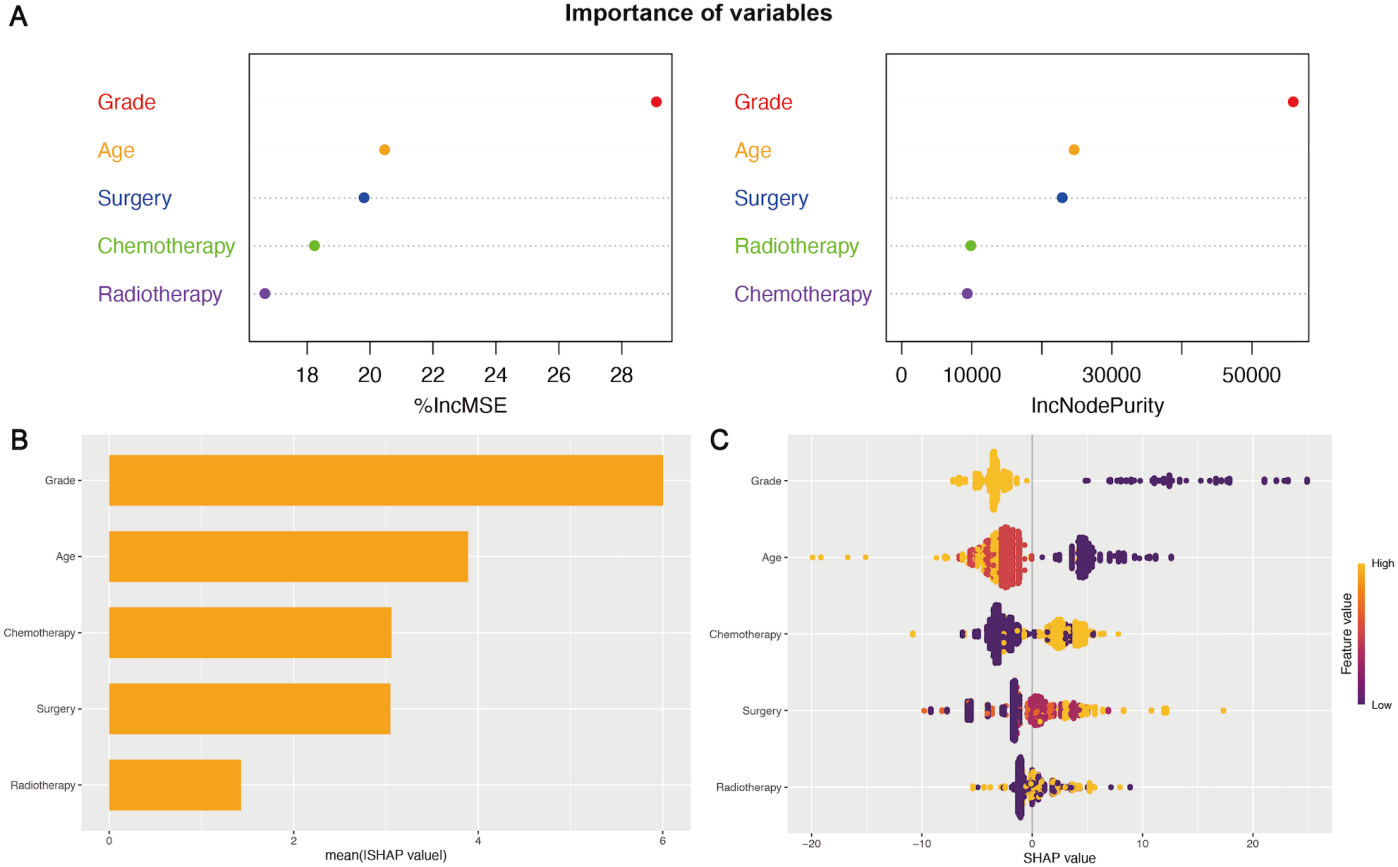
The importance of variables in the prognostic nomogram. **(A)** The IncMSE (left) and IncNodePurity (right) based on the random forest model emphasized the notable influence of variables on the model’s predictive accuracy. **(B, C)** SHAP summary plots of variables in the nomogram. The ranking of variables’ importance according to the mean SHAP value in machine learning **(B)**. A wider spread of points in the beeswarm plot indicated a stronger influence of the variables in the model **(C)**.

The importance of five key variables derived from the Model 3 (the LASSO model) was further evaluated using the random forest model and machine learning (**Figure 4**). The mean square error (%IncMSE) and node purity (IncNodePurity) values were calculated to assess the model based on random forest (**Figure 4A**). Higher %IncMSE values reflected notable enhancements, and IncNodePurity quantified the improvement in node purity with the introduction of a feature, highlighting the critical importance of WHO grade and age in enhancing the model’s predictive accuracy. Moreover, a machine learning approach was utilized to interpret the impact of variables on the model’s predictive accuracy. The mean Shapley additive explanations (SHAP) scores of five key variables highlighted their significant contributions to predictive effectiveness (**Figures 4B, C**). According to the above importance analysis of the variables, age is still a very important variable affecting survival and the predictive power of the nomogram, although the variable was screened from the elderly population.

### Development and validation of prognostic nomograms

**Figure 5:**
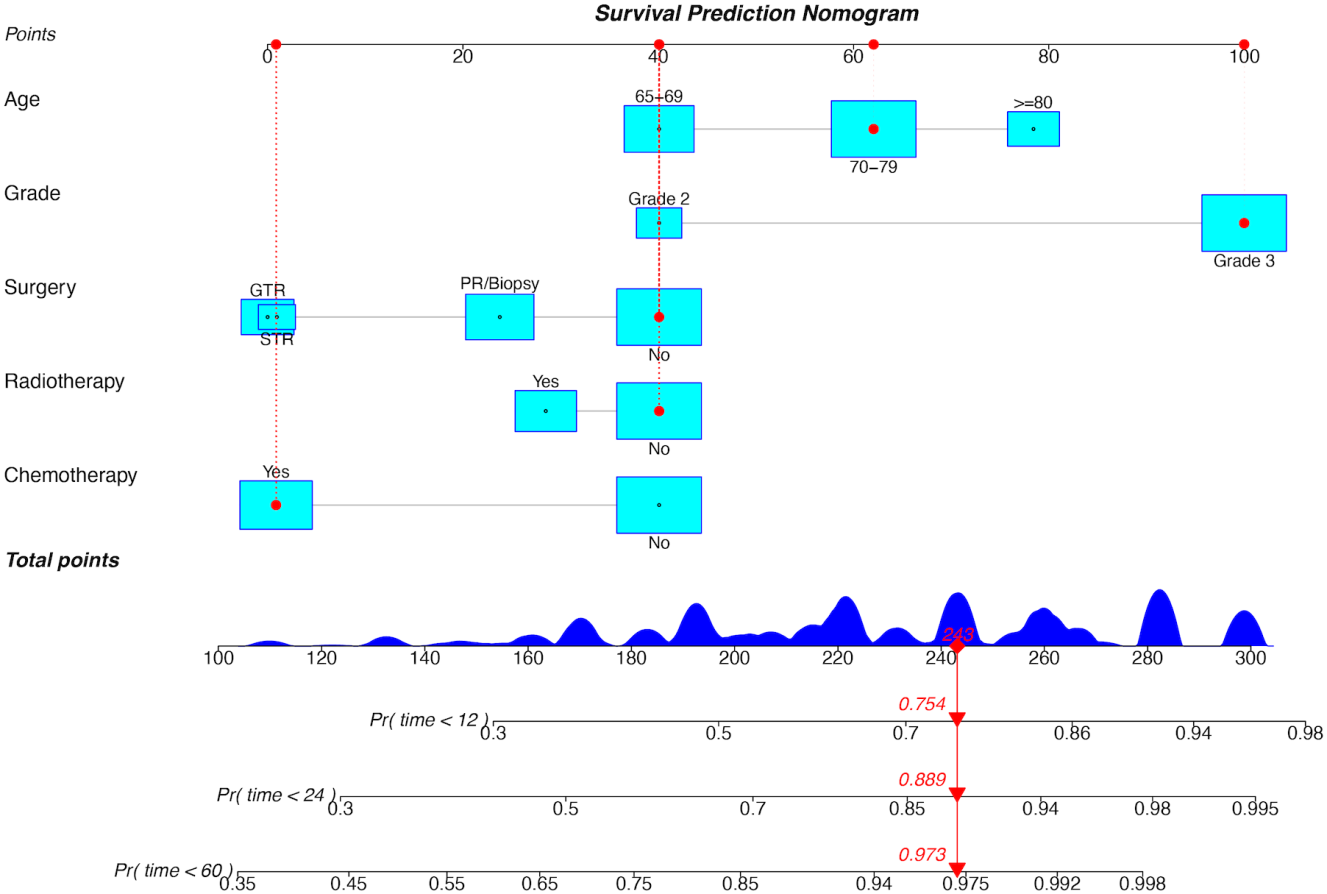
Prognostic nomogram for OS in the training cohort. GTR, gross total resection; STR, subtotal resection; PR, partial resection; Pr, probability.

**Figure 6:**
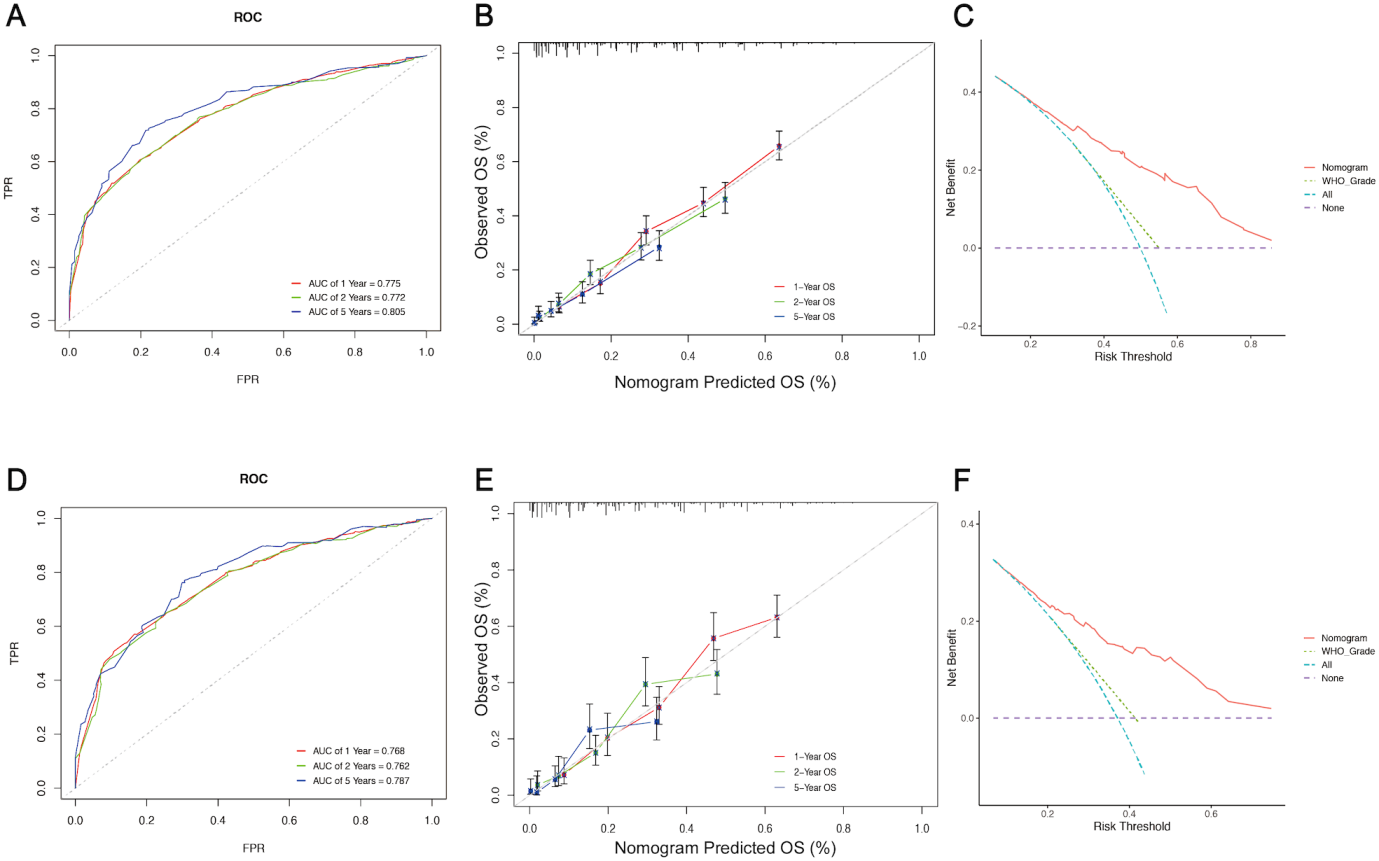
Assessment and validation of the prognostic nomogram for OS. ROC curves **(A)**, calibration curves **(B)**, and DCA curves **(C)** of the nomogram in the training cohort. ROC curves **(D)**, calibration curves **(E)**, and DCA curves **(F)** of the nomogram in the validation cohort. ROC curve, Receiver operating characteristic curve; DCA, Decision Curve Analysis; FPR, False positive rate; TPR, True positive rate.

The five independent prognostic predictors (age, WHO grade, surgery, radiotherapy, and chemotherapy) were pooled to construct prognostic nomograms that can quantitatively predict the 1-year, 2-year, and 5-year OS (**Figure 5**) and CSS (**Supplementary Figure 3**). Internal and external validation of these prognostic nomograms was conducted to assess their discrimination ability and reliability in the training and validation cohorts. The C-indexes for the nomogram for OS were 0.708 (95% CI, 0.693-0.723) and 0.694 (95% CI, 0.674-0.714) for internal and external validation, respectively. Similarly, the C-index values for the nomogram for CSS were 0.704 (95% CI, 0.687-0.721) and 0.680 (95% CI, 0.660-0.700) for internal and external validation, respectively. These C-index and AUC values indicated the relatively good predictive ability and applicability of the nomograms (**Table 4**). Additionally, the ROC curves and calibration plots for 1-, 2-, and 5-year OS demonstrated good predictive performance and consistency across both the training and validation cohorts. Furthermore, DCA curves revealed that the prognostic nomograms for OS and CSS offered a greater net benefit than did the WHO grade (**Figure 6**; **Supplementary Figure 4**).

**Table 4:** The C-index and AUC values of the prognostic nomograms for OS and CSS in the training and validation cohorts.

Groups	Training cohort	Validation cohort
C-index (95% CI)		
OS	0.710 (0.695-0.725)	0.700 (0.680-0.720)
CSS	0.705 (0.687-0.723)	0.690 (0.670-0.710)
AUC		
1-year OS	0.775	0.761
2-year OS	0.772	0.769
5-year OS	0.805	0.788
1-year CSS	0.768	0.738
2-year CSS	0.762	0.759
5-year CSS	0.787	0.774

AUC, Area under the curve; CI, confidence interval; C-index, Harrell’s concordance index; OS, overall survival; CSS, cancer-specific survival.

### Risk stratification system and web-based dynamic nomogram

**Figure 7:**
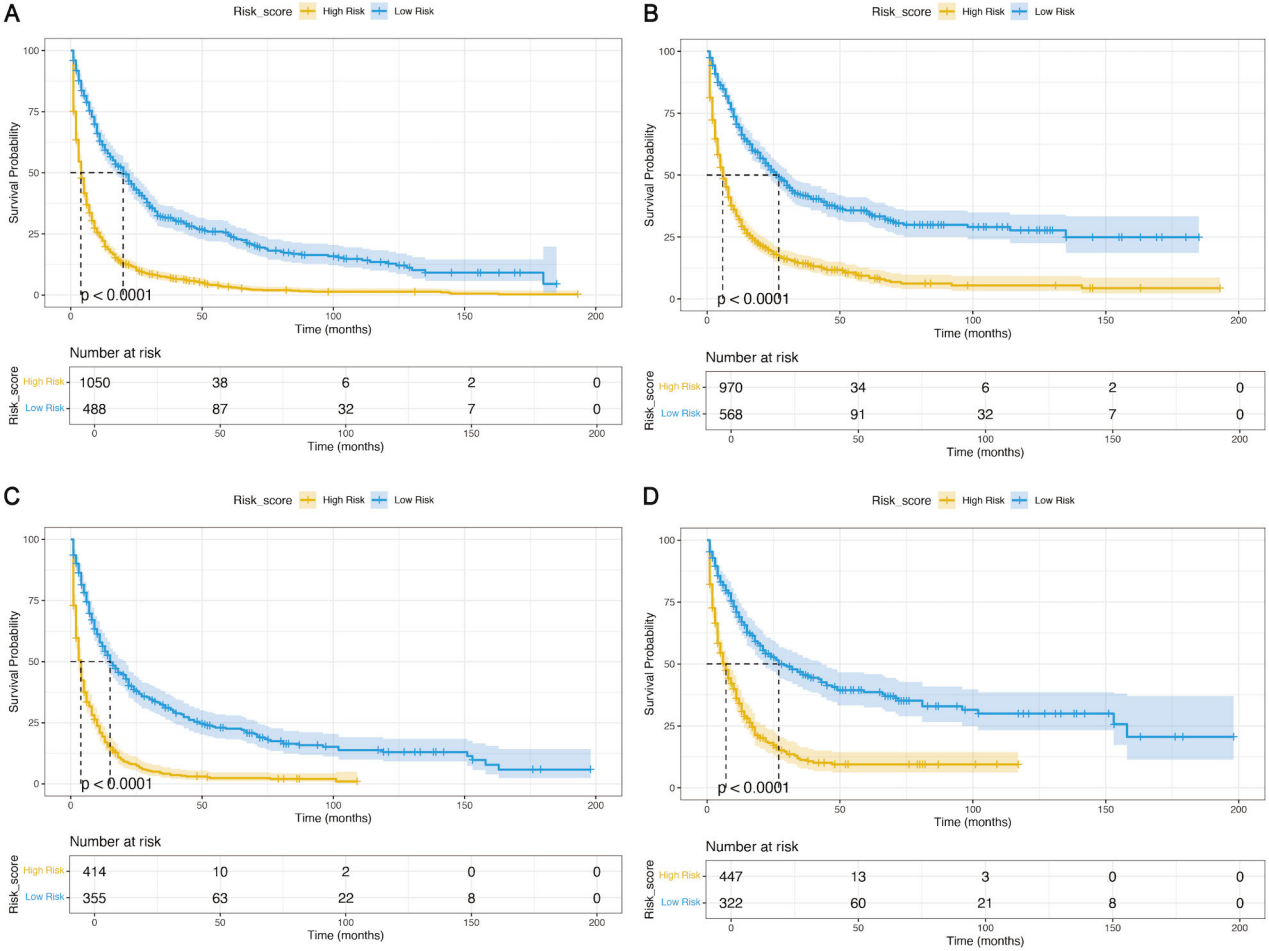
K−M analyses were used to determine the impact of the risk stratification system based on the prognostic nomograms on OS and CSS. **(A, B)** Based on the nomograms on OS **(A)** and CSS **(B)** in the training cohort. **(C, D)** Based on the nomogram on OS **(C)** and CSS **(D)** in the validation cohort.

**Figure 8:**
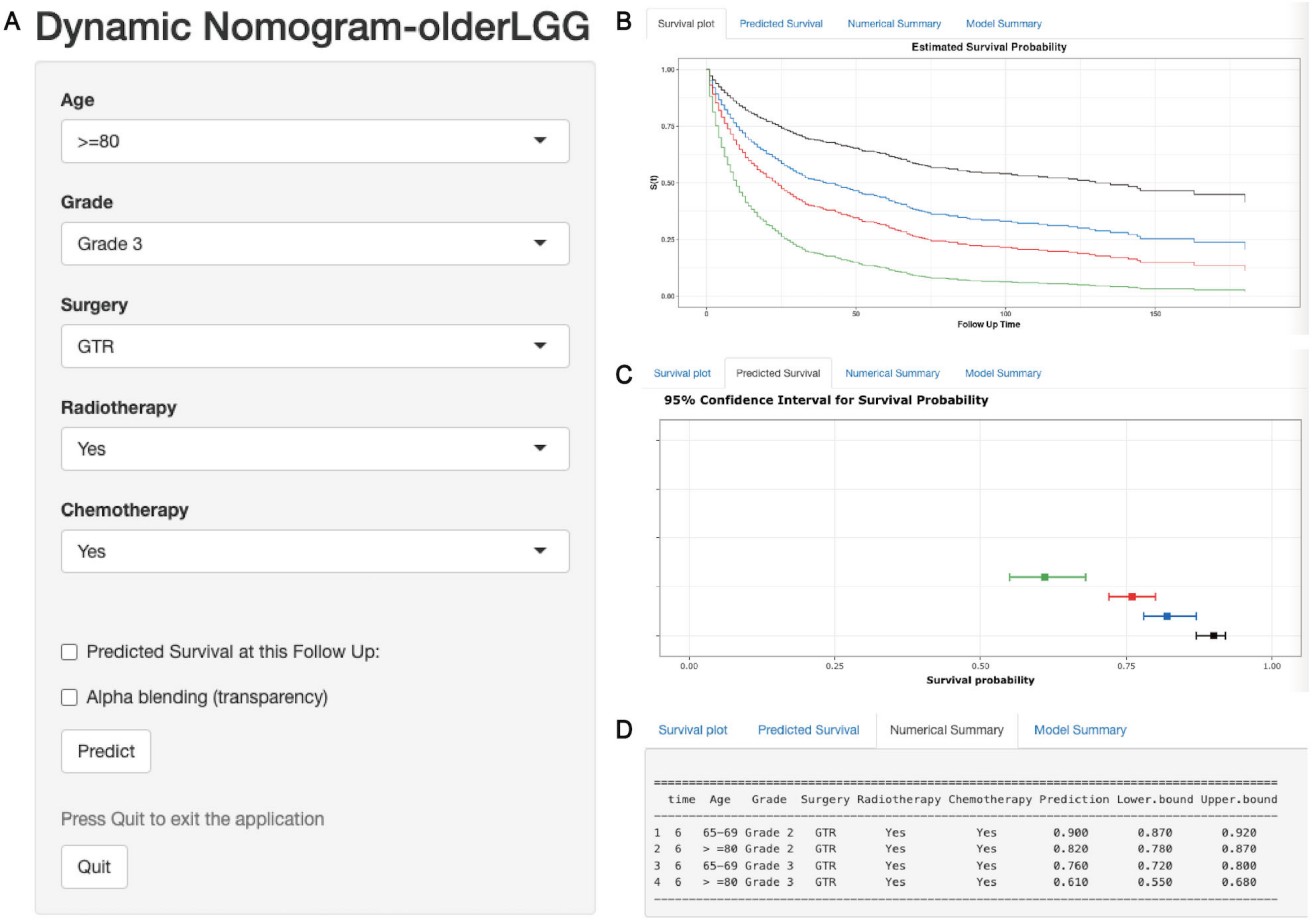
A web-based dynamic nomogram for predicting OS of older patients with LGGs. **(A)** The nomogram incorporated a panel of independent prognostic factors for the prediction of survival outcomes. **(B)** A survival plot depicting OS based on different clinical features. **(C)** The graphical summary included line segments indicating the 95% confidence intervals (CIs) for the estimated survival probability. **(D)** The numerical summary provided detailed information on the variable features and exact predictive values of OS.

Elderly patients with LGGs were categorized into two subgroups of mortality risk according to the median total score of the nomogram: the low-risk subgroup and the high-risk subgroup. Furthermore, the K−M survival curves revealed a significant difference in survival between these two groups in the training and validation cohorts, suggesting that the prognostic nomograms had significant clinical value for OS and CSS prediction (**Figure 7**). Additionally, a web-based dynamic nomogram was constructed to facilitate the clinical application of the predictive model. By inputting the corresponding information of the independent prognostic factors, the survival probability of elderly patients with LGGs could be quickly predicted. This dynamic nomogram is available on the following website: https://allenscu.shinyapps.io/olderLGG/. Moreover, we present an example of an application for calculating the OS survival probability according to different parameters of various variables (**Figure 8**).

## Discussion

LGGs are characterized by relatively slow growth, infiltrative behavior, and progression and constitute approximately 15% of all adult brain glial tumors (3). In contrast, glioblastoma (GBM) is more prevalent in elderly patients, whereas LGGs are relatively uncommon in the elderly population (9, 17, 18). Previously, Gittleman et al. (15) constructed a survival nomogram for patients with LGGs that included mainly patients <65 years old. Moreover, this prognostic nomogram showed some differences in prediction efficiency among different races when it was validated in a large-scale Asian cohort (19). Given the limited inclusion of older data in prior studies, this study identified independent prognostic factors affecting survival and constructed prognostic nomograms for predicting the OS and CSS probabilities of elderly patients with LGGs at the individual patient level.

This study included a total of 13 variables and the optimal variables were screened by using different methods (Cox regression and LASSO-Cox regression analysis). First, we identified four independent prognostic factors (age, WHO grade, surgery, and chemotherapy, included in the Model 2) that impact OS and CSS using full Cox regression. Additionally, LASSO regression is a novel method for variable screening that involves applying a penalized regression on all variable coefficients, which can exclude the coefficients of relatively unimportant independent variables from the model (20–24). Gu et al. (23) used two variable screening methods in the validation cohort of postoperative pancreatic fistula after pancreatoduodenectomy, and the results revealed that the Lasso-logistic regression model included the optimal independent variables and had better predictive ability than the full logistic regression model. Thus, we used this novel method (LASSO regression analysis based on the Cox model) to screen five variables (age, WHO grade, surgery, radiotherapy, and chemotherapy included in Model 3), which included one more radiotherapy variable than the above Cox regression. A comparison of Model 2 and Model 3 revealed that the prediction ability of Model 3 was better than that of Model 2. Moreover, the five variables in the Model 3 were more closely related to the clinical management of LGG patients. Finally, 5 variables in the Model 3 were included to construct prognostic nomograms.

According to the importance analysis of the five variables, the random forest model and SHAP machine learning analysis revealed the significant impact of WHO grade and age on the predictive accuracy of the model, facilitating the interpretation of variable influence in the nomogram (25–27). Despite emerging studies focusing on elderly individuals (aged over 65 years), age still emerged as a significant independent prognostic factor of OS and CSS, with older patients exhibiting poorer survival outcomes, mirroring findings in GBM patients spanning various age groups (28–32). In addition, the other three variables (surgery, radiotherapy, and chemotherapy) also play important roles in affecting the survival and predictive performance of the model. Surgical resection is considered the primary treatment for LGGs, and GTR of the tumor is associated with better survival outcomes than STR, PR, or biopsy in diffuse gliomas, regardless of grade (33–35). In our study, we found that GTR was significantly associated with longer OS in grade 2 or 3 elderly patients with LGGs. Previous evidence has also supported the importance of maximal safe resection and recommended GTR for elderly patients with LGG (35). However, notably, the treatment protocols and diagnostic criteria have evolved over a relatively long span (2000 ~ 2016). In this study, a greater proportion of patients without surgical intervention (45.4%) and a lower proportion of patients receiving adjuvant therapy (54.5%) had a generally poorer prognosis observed in this study, which might have impacted the study conclusions and should be interpreted with caution. Furthermore, postoperative adjuvant therapy has been shown to prolong the OS of LGG patients, especially those with high-risk gliomas (36–39). In our study, although molecular and risk feature data for elderly LGG patients were lacking, subgroup analysis demonstrated that GTR/STR combined with adjuvant therapy, including radiotherapy and chemotherapy, led to better survival outcomes in grade 3 gliomas. However, in grade 2 gliomas, GTR/STR without adjuvant therapy, especially radiotherapy, provided the best survival benefit. These findings align with those of previous studies and strengthen the evidence on the postoperative management of LGGs in elderly patients (38).

Finally, five key variables screened by LASSO-Cox regression analysis were utilized to construct prognostic nomograms for OS and CSS. A nomogram is a visual representation of a predictive model commonly utilized to predict disease outcomes, including survival for various diseases (25, 40–42). To the best of our knowledge, several clinical prognostic models have been developed for LGGs based on clinical and genetic signatures (43, 44). Moreover, predictive models (43) based on special gene and metabolic signatures restrict their general application in the clinic due to the limited clinical detection of specific genetic features. However, there is a lack of clinically prognostic nomograms specifically designed for the elderly population with LGGs. Compared with the previous survival prediction model for patients with LGGs with an average age of approximately 40 years (44), our nomogram model was unable to include two variables (IDH molecular status and preoperative neurological deficit) due to database limitations. However, we included two additional independent prognostic variables (radiotherapy and chemotherapy) in the nomogram model tailored to elderly LGG patients using the largest sample size, and the performance of the prognostic nomograms demonstrated relatively good predictive ability for survival in these elderly individuals. DCA curves were utilized to further evaluate the clinical effectiveness of the nomogram models (27, 42). DCA curves indicated that the prognostic nomograms provided a superior net benefit compared with the WHO grading system.

Moreover, a risk stratification system based on prognostic nomograms was developed for elderly patients with LGGs, which could effectively stratify these patients into low-risk and high-risk subgroups. This risk stratification system can assist clinicians in determining treatment strategies and could effectively avoid wasting medical resources. Moreover, a web-based dynamic nomogram for elderly LGG patients has been developed to facilitate its use in the clinic and could help clinicians quickly develop personalized decision-making strategies (42, 45).

This retrospective study has several limitations. First, considering the number of included patients over a long period, changes in glioma classification guidelines may impact overall survival outcomes and prognostic factors for patients with LGGs (46). For example, the minority of histological LGGs with molecular features of GBMs were diagnosed as grade 4 gliomas according to the latest guidelines (46). However, crucial variables and molecular markers, such as the Karnofsky Performance Scale (KPS) score, IDH mutation status, and 1p/19q codeletion, were not recorded in the SEER database and thus cannot be incorporated into the model. Finally, given that the training and validation datasets for the predictive models were derived from the SEER database, it is imperative to conduct future validations of the nomogram’s performance and reliability across different databases.

In summary, this study identified five independent prognostic factors using LASSO-Cox regression with a larger sample size for elderly patients with LGGs from the SEER database. By integrating these prognostic factors, novel prognostic nomograms were constructed to predict the 1-year, 2-year, and 5-year probabilities of OS and CSS. Moreover, the construction of the risk stratification system and web-based dynamic nomogram further facilitated its use in the clinic and could help clinicians quickly develop clinical assessments and personalized decision-making strategies.

## Data Availability

The original contributions presented in the study are included in the article/**Supplementary Material**. Further inquiries can be directed to the corresponding authors.
