# Supplementary material for: Variable screening and model construction for prognosis of elderly patients with lower-grade gliomas based on LASSO-Cox regression: a population-based cohort study
Source: Front Immunol. 2024 Sep 11;15:1447879. doi: 10.3389/fimmu.2024.1447879 (PMC11422072; doi:10.3389/fimmu.2024.1447879)
Supplement: Supplementary file 1 [file DataSheet1.pdf]

Supplementary Material

1 Supplementary Data

Supplemental Figure 1. Kaplan-Meier analysis determining the impact of treatment regimens on OS in grade 2 patients. (A) Stratified by surgery, (B) Stratified by radiotherapy, (C) Stratified by chemotherapy, (D) Stratified by adjuvant therapy, (E) Stratified by treatment regimens.

Supplemental Figure 2. Kaplan-Meier analysis determining the impact of treatment regimens on OS in grade 3 patients. (**A**) Stratified by surgery, (**B**) Stratified by radiotherapy, (**C**) Stratified by chemotherapy, (**D**) Stratified by adjuvant therapy, (**E**) Stratified by treatment regimens.

Supplemental Figure 3. Prognostic nomogram for CSS in the training cohort.

Supplemental Figure 4. Assessment and validation of the predictive nomogram for CSS. ROC curves (**A**), calibration curves (**B**), and DCA curves (**C**) of the nomogram in the training cohort. ROC curves (**D**), calibration curves (**E**), and DCA curves (**F**) of the nomogram in the validation cohort.

Supplemental Table 1. Multivariate Cox regression analyses of OS and CSS in the training cohort.

2 Supplementary Figures and Tables

2.1 Supplementary Figures

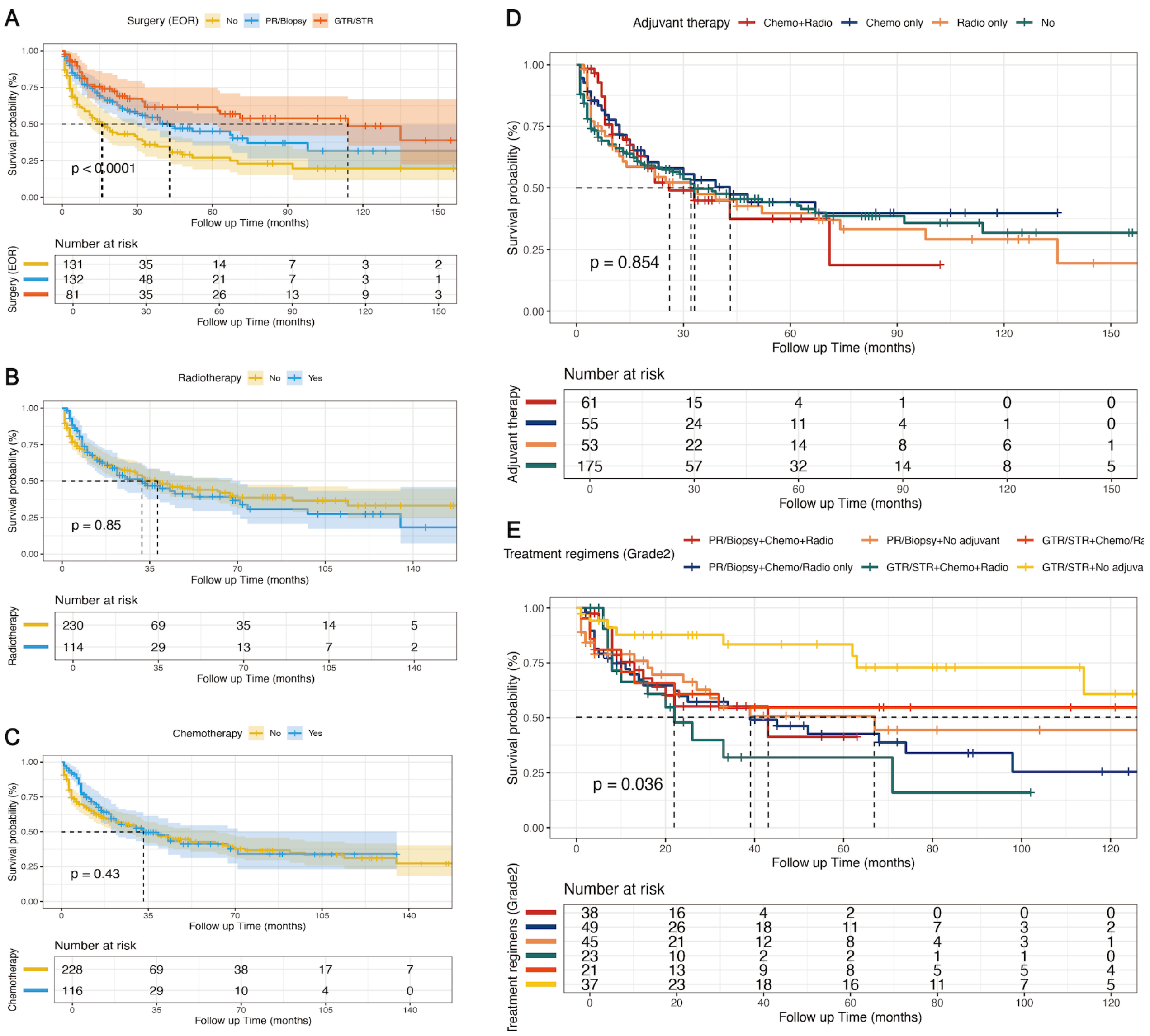

Supplemental Figure 1. Kaplan-Meier analysis determining the impact of treatment regimens on OS in grade 2 patients. (**A**) Stratified by surgery, (**B**) Stratified by radiotherapy, (**C**) Stratified by chemotherapy, (**D**) Stratified by adjuvant therapy, (**E**) Stratified by treatment regimens.

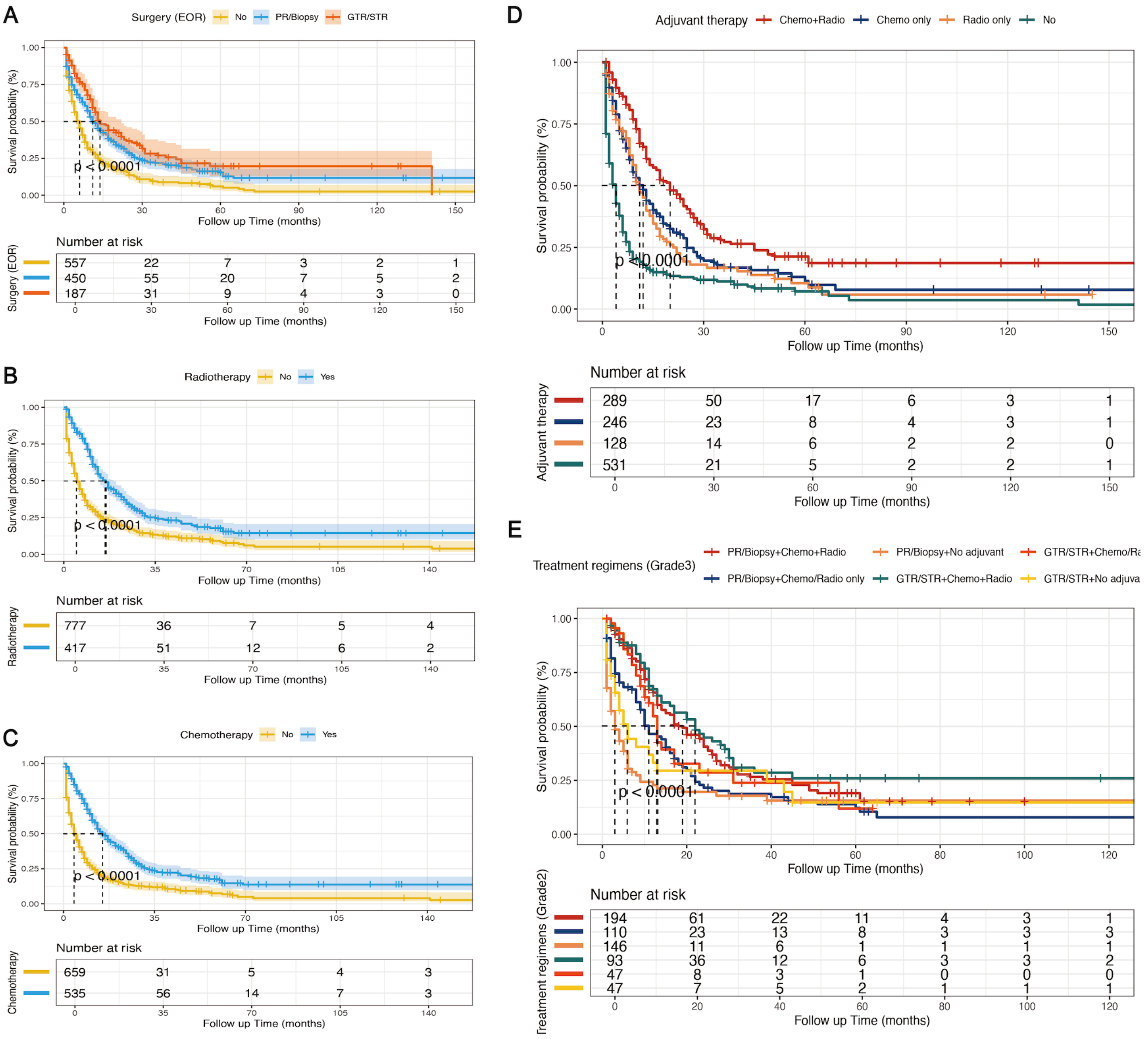

Supplemental Figure 2. Kaplan-Meier analysis determining the impact of treatment regimens on OS in grade 3 patients. (**A**) Stratified by surgery, (**B**) Stratified by radiotherapy, (**C**) Stratified by chemotherapy, (**D**) Stratified by adjuvant therapy, (**E**) Stratified by treatment regimens.

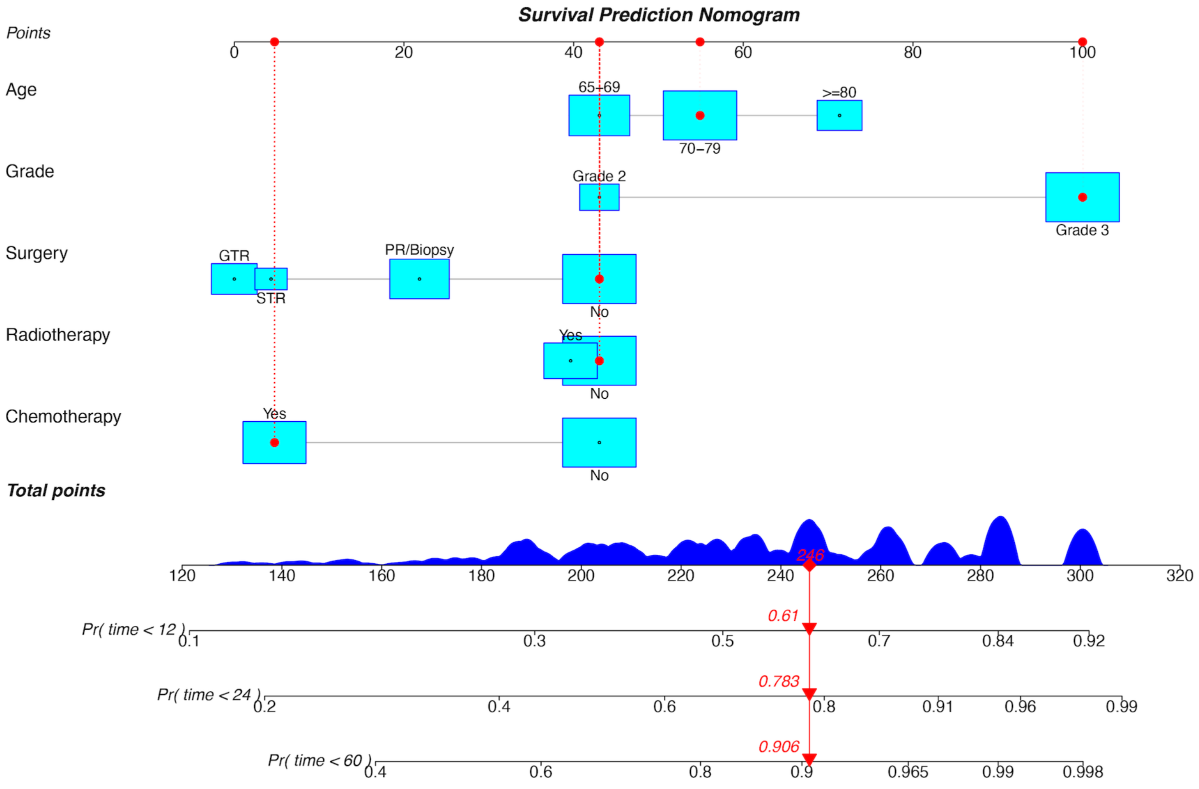

Supplemental Figure 3. Prognostic nomogram for CSS in the training cohort.

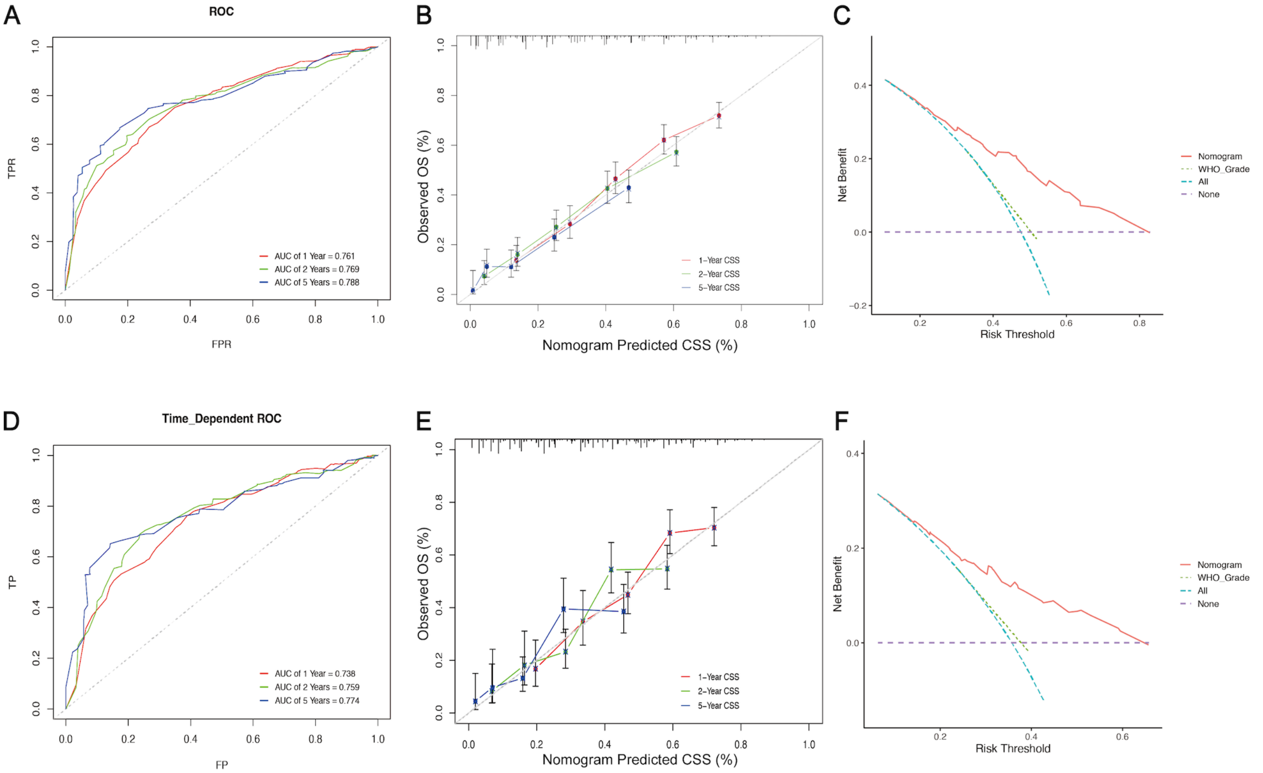

Supplemental Figure 4. Assessment and validation of the predictive nomogram for CSS. ROC curves (**A**), calibration curves (**B**), and DCA curves (**C**) of the nomogram in the training cohort. ROC curves (**D**), calibration curves (**E**), and DCA curves (**F**) of the nomogram in the validation cohort.

2.2 Supplementary Tables

Supplemental Table 1. Multivariate Cox regression analyses of OS and CSS in the training cohort.

Variables	OS			CSS		
	HR	95%CI	*P* value	HR	95%CI	*P* value
Age at diagnosis (years)						
≥80/70-79/65-69	1.381	1.090-1.750	**0.007**	**1.203**	**1.050-1.410**	**0.01**
Tumor side						
(Bilateral/Midline)/Unila	1.664	0.892-3.102	0.109	1.829	0.851-3.930	0.122
Tumor site						
Non-frontal/Frontal lobe	1.176	0.851-1.624	0.325	1.169	0.780-1.754	0.449
WHO Grade						
Grade III/II	2.723	1.865-3.976	**<0.001**	3.306	2.033-5.375	**<0.001**
Surgery						
(GTR/STR)/(PR/Biopsy)	0.837	0.741-0.945	**0.004**	0.857	0.739-0.993	**0.039**
Radiotherapy						
Yes/No	0.958	0.653-1.408	0.829	0.962	0.606-1.528	0.870
Chemotherapy						
Yes/No	0.560	0.398-0.788	**0.001**	0.459	0.298-0.708	**<0.001**

OS: overall survival; CSS: cancer-specific survival; HR, hazard ratio; CI, confidence interval.
